# Associations of Variants in *CHRNA5/A3/B4* Gene Cluster with Smoking Behaviors in a Korean Population

**DOI:** 10.1371/journal.pone.0012183

**Published:** 2010-08-16

**Authors:** Ming D. Li, Dankyu Yoon, Jong-Young Lee, Bok-Ghee Han, Tianhua Niu, Thomas J. Payne, Jennie Z. Ma, Taesung Park

**Affiliations:** 1 Department of Psychiatry and Neurobehavioral Sciences, University of Virginia, Charlottesville, Virginia, United States of America; 2 Interdisciplinary Program in Bioinformatics, Seoul National University, Seoul, Korea; 3 Center for Genome Science, National Institute of Health, Seoul, Korea; 4 Department of Otolaryngology and Communicative Sciences, University of Mississippi Medical Center, Jackson, Mississippi, United States of America; 5 Department of Public Health Sciences, University of Virginia, Charlottesville, Virginia, United States of America; 6 Department of Statistics, College of Natural Science, Seoul National University, Seoul, Korea; National Institutes of Health, United States of America

## Abstract

Multiple genome-wide and targeted association studies reveal a significant association of variants in the *CHRNA5*-*CHRNA3*-*CHRNB4* (*CHRNA5/A3/B4*) gene cluster on chromosome 15 with nicotine dependence. The subjects examined in most of these studies had a European origin. However, considering the distinct linkage disequilibrium patterns in European and other ethnic populations, it would be of tremendous interest to determine whether such associations could be replicated in populations of other ethnicities, such as Asians. In this study, we performed comprehensive association and interaction analyses for 32 single-nucleotide polymorphisms (SNPs) in *CHRNA5/A3/B4* with smoking initiation (SI), smoking quantity (SQ), and smoking cessation (SC) in a Korean sample (N = 8,842). We found nominally significant associations of 7 SNPs with at least one smoking-related phenotype in the total sample (SI: P = 0.015∼0.023; SQ: P = 0.008∼0.028; SC: P = 0.018∼0.047) and the male sample (SI: P = 0.001∼0.023; SQ: P = 0.001∼0.046; SC: P = 0.01). A spectrum of haplotypes formed by three consecutive SNPs located between rs16969948 in *CHRNA5* and rs6495316 in the intergenic region downstream from the 5′ end of *CHRNB4* was associated with these three smoking-related phenotypes in both the total and the male sample. Notably, associations of these variants and haplotypes with SC appear to be much weaker than those with SI and SQ. In addition, we performed an interaction analysis of SNPs within the cluster using the generalized multifactor dimensionality reduction method and found a significant interaction of SNPs rs7163730 in *LOC123688*, rs6495308 in *CHRNA3*, and rs7166158, rs8043123, and rs11072793 in the intergenic region downstream from the 5′ end of *CHRNB4* to be influencing SI in the male sample. Considering that fewer than 5% of the female participants were smokers, we did not perform any analysis on female subjects specifically. Together, our detected associations of variants in the *CHRNA5/A3/B4* cluster with SI, SQ, and SC in the Korean smoker samples provide strong evidence for the contribution of this cluster to the etiology of SI, ND, and SC in this Asian population.

## Introduction

There are about 1.3 billion smokers worldwide, and the mortality burden from tobacco use has been estimated to exceed 6 million annually [1]. In Korea, male smoking prevalence is among the highest in the world [2]. In 2000, the prevalence was estimated to be 68% among men and 3% among women [2]. The number of deaths attributable to smoking-related diseases in Korea is about 35,000 each year, and the economic loss from premature death from smoking-related diseases exceeded 3 trillion won (approximately US $2.5 billion) in the year 2000 [3].

Nicotine is the psychoactive substance in tobacco that causes addiction. Many of those who want to quit smoking do not seek treatment but are unable to quit on their own [4]. Evidence for moderate heritabilities of smoking initiation (SI), nicotine dependence (ND), and smoking cessation (SC) has led to intensive efforts to identify susceptibility loci for these complex behavioral phenotypes [5], [6], [7], [8].

The psychopharmacologic effects of nicotine are mediated primarily by functionally diverse neuronal nicotinic acetylcholine receptors (nAChRs), a family of ligand-gated ion channels widely distributed in the brain [9], [10], [11]. Because of their unique functions, genes encoding various nAChR subunits have been proposed as plausible candidates for genetic studies of ND. Several subunit genes have been investigated for associations with ND as well as other smoking-related behaviors in human subjects (for reviews, see [12], [13]).

Initially, Saccone *et al*. [14] reported associations of multiple single nucleotide polymorphisms (SNPs) in the *CHRNA5/A3/B4* gene cluster with ND, with the smallest P value of 0.00064 for rs16969968 (D398N) in exon 5 of *CHRNA5*. However, the significance of these results did not survive correction for multiple testing. Shortly after the initial publication, rs1051730 (Y215Y) in exon 5 of *CHRNA3* was reported to be significantly associated at a genome-wide level with smoking quantity (SQ) [15] and lung cancer (LC) [15], [16], [17]. Several other genome-wide and candidate gene-based association studies provided further evidence for the association of variants of the *CHRNA5/A3/B4* cluster with various nicotine-related behaviors [14], [18], [19], [20], [21], [22], [23], [24], [25]. In contrast, other studies have failed to reveal a significant association of this gene cluster with ND or other smoking-related phenotypes [26], [27].

Considering that (1) the participants in most of these studies were primarily European Americans or of European origin, with the exception of two studies on African Americans [18], [28], and (2) there are distinct differences in linkage disequilibrium (LD) patterns across different ethnic populations [12], it is of tremendous interest to determine whether variants in this gene cluster play any role in the etiologies of smoking behavioral phenotypes in other ethnic groups. Thus, the major objective of this study was to test for such genetic effects in a large population-based Korean sample.

## Results

### Description of KARE sample in its relation to smoking behaviors

Of the 8,842 subjects, 4,205 were recruited from Ansung and 4,637 from Ansan, Korea. Their average ages were 55.6±8.74 (standard deviation; SD) and 49.1±7.86 years, respectively. Although 52.7% of the participants (N = 4,659) were female, only 4.93% of these were considered either former (1.34%), light (1.29%), or habitual (2.30%) smokers. In contrast, 80.62% of the male subjects were smokers, with 31.05% being former smokers, 4.78% light smokers, and 44.79% habitual smokers. For those habitual smokers, the average number of cigarettes smoked per day (CPD) was 19.51±8.74 for male and 11.93±7.28 for female smokers. A detailed description of the characteristics of all subjects is presented in Table 1.

**Table 1:** Demographic characteristics of study subjects.

Category	Sub-Category	Ansung	Ansan	Total
Sample Size (N)		4,205	4,637	8,842
Male/female (%)		1,809 (43)/2,396 (57)	2,374 (51.2)/2,263 (48.8)	4,183 (47.3)/4,659 (52.7)
Mean age (years)±SD		55.60±8.74	49.08±7.86	52.22±8.92
Smoking status (Total sample)	Never smoked	2,492	2,651	5,143
	Mean CPD for “Former Smokers”± SD (N)	18.88±11.88(512)	19.11±12.14(842)	19.02±12.04(1,354)
	Number of occasional smokers	155	103	258
	Mean CPD for “Habitual Smokers” ± SD (N)	19.33±8.41(950)	18.91±9.20(1,020)	19.11±8.83(1,970)
Smoking status (Male sample)	Never smoked	306	501	807
	Mean CPD for “Former Smokers” ± SD (N)	19.60±11.76(481)	19.52±12.06(812)	19.55±11.94(1,293)
	Number of occasional smokers	118	81	199
	Mean CPD for “Habitual Smokers” ± SD (N)	19.81±8.20(887)	19.23±9.19(978)	19.51±8.74(1,865)
Smoking status (Female sample)	Never smoked	2,186	2,150	4,336
	Mean CPD for “Former Smokers” ± SD (N)	6.35±4.96(31)	7.07±7.53(30)	6.72±6.35(61)
	Number of occasional smokers	37	22	59
	Mean CPD for “Habitual Smokers” ± SD (N)	12.42±8.30(63)	11.18±5.34(42)	11.93±7.28(105)

### Individual SNP-based association analysis

Among the 36 SNPs genotyped for the 15q24-15q25.1 region in *CHRNA5/A3/B4*, only 32 had a minor allele frequency (MAF) of >0.01. Considering the differences in LD patterns of the region across multiple ethnic samples and to have a better understanding of the LD landscape within this region in the Korean vs. the other ethnic populations, Table S1 provides a detailed list of all genotyped SNPs for the total and male samples. However, only those 32 SNPs with MAFs >0.01 were used in the association analyses for the three smoking-related phenotypes.

Associations of individual SNPs with the three phenotypes were determined with the PLINK program [29], and the results are shown in Table 2. Altogether, we found 7 SNPs that had nominally significant associations with at least one smoking-related phenotype in either the total or the male sample. In the total sample, we found that the A allele of rs951266 in *CHRNA5* was nominally significantly associated with SI (P = 0.023; odds ratio [OR]  = 1.32; 95% confidence interval [CI]: = 1.04, 1.67) and SQ (P = 0.008; OR = 1.48; 95% CI: 1.11, 1.98), and the G allele of rs11072768 in *CHRNB4* was nominally significantly associated with SI (P = 0.015; OR = 1.14; 95% CI: 1.03, 1.27), SQ (P = 0.028; OR = 1.17; 95% CI: 1.02, 1.34), and SC (P = 0.018; OR = 1.16; 95% CI: 1.03, 1.31). Furthermore, the associations of SNPs rs8043123 (C), rs4887077 (T), and rs11638372 (T) with SQ and the association of rs2869550 (C) with SC reached nominal significance.

**Table 2:** P values for SNPs significantly associated with at least one smoking-related phenotype and their corresponding odds ratios and 95% confidence intervals under the additive and dominant model.

			Total Sample						Male Sample					
			SI		SQ		SC		SI		SQ		SC	
Gene	dbSNP ID	Risk Allele	P	OR(95% CI)	P	OR(95% CI)	P	OR(95% CI)	P	OR(95% CI)	P	OR(95% CI)	P	OR(95%CI)
*CHRNA5*	rs951266	A	**0.023^a^**	1.32(1.04, 1.67)	**0.008^a^**	1.48(1.11, 1.98)	0.405^a^	1.14(0.84, 1.55)	**0.006^a^**	1.46(1.11, 1.91)	**0.001^a^**	1.71(1.24, 2.34)	0.351^a^	1.16(0.85, 1.60)
*CHRNA3*	rs6495308	T	0.150^a^	1.06(0.98, 1.16)	0. 110^a^	1.09(0.98, 1.21)	0.287^a^	1.06(0.95, 1.19)	**0.023^a^**	1.11(1.02, 1.22)	**0.046^a^**	1.12(1.00, 1.26)	0.230^a^	1.07(0.96, 1.20)
*CHRNB4*	rs11072768	G	**0.015^d^**	1.14(1.03, 1.27)	**0.028** ^d^	1.17(1.02, 1.34)	**0.018^a^**	1.16(1.03, 1.31)	**0.001^d^**	1.22(1.08, 1.37)	**0.016^a^**	1.16(1.03, 1.31)	**0.010^a^**	1.18(1.04, 1.34)
*Intergenic region*	rs8043123	C	0.329^d^	0.93(0.80, 1.08)	**0.022^d^**	0.84(0.73, 0.98)	0. 218^a^	0.94(0.85, 1.04)	0.422^a^	1.05(0.94, 1.17)	0.092^d^	0.88(0.75, 1.02)	0. 256^a^	0.94(0.85, 1.04)
*Intergenic region*	rs4887077	T	0.079^a^	1.39(0.96, 2.01)	**0.022^a^**	1.51(1.06, 2.14)	0.575^d^	0.90(0.63, 1.30)	0.063^a^	1.58(0.98, 2.55)	**0.008^a^**	1.68(1.15, 2.47)	0.552^d^	0.89(0.61, 1.30)
*Intergenic region*	rs2869550	C	0.554^d^	1.03(0.92, 1.16)	0.659^d^	1.03(0.89, 1.19)	**0.047^d^**	1.16(1.00, 1.35)	0.538^d^	1.04(0.92, 1.18)	0.819^d^	1.02(0.87, 1.19)	0.079^d^	1.15(0.98, 1.34)
*Intergenic region*	rs11638372	T	0.102^a^	1.36(0.94, 1.96)	**0.027^a^**	1.48(1.05, 2.09)	0.645^d^	0.92(0.64, 1.32)	0.081^a^	1.52(0.95, 2.43)	**0.010^a^**	1.65(1.13, 2.41)	0.623^d^	0.91(0.63, 1.32)

(1) OR  =  odds ratio; CI  =  confidence interval; SI  =  smoking initiation; SC  =  smoking cessation, SQ  =  smoking quantity. (2) Significant associations at the 0.05 level before Bonferroni correction for multiple testing are given in bold and those significant after Bonferroni correction for multiple testing are in bold and underlined (corrected P value at a 0.05 significance level is 0.0016 ( = 0.05/32). (3) Superscripts indicate the genetic model used in the analysis: a  =  additive and d  =  dominant. (4) For each sample, age, sex, and area were used as covariates.

In the male sample, the G allele of rs11072768 in *CHRNB4* was nominally significantly associated with SI (P = 0.001; OR = 1.22; 95% CI: 1.08, 1.37), SQ (P = 0.016; OR = 1.16; 95% CI: 1.03, 1.31), and SC (P = 0.01; OR = 1.18; 95% CI: 1.04, 1.34), and rs951266 (A) in *CHRNA5* and rs6495308 (T) in *CHRNA3* were nominally significantly associated with SI and SQ, respectively. In addition, we found that SNPs rs4887077 (T) and rs11638372 (T) in the intergenic region downstream from the 5′ end of *CHRNB4* were nominally significantly associated with SQ. Of these associations, only that of rs951266 in *CHRNA5* with SQ and rs11072768 in *CHRNA4* with SI remained significant after correction for multiple testing.

We did not perform the association analysis on the female sample because fewer than 5% of these subjects smoked, such that the sample was too small to derive any meaningful conclusions.

### Haplotype block structure and LD analysis

**Figure 1:**
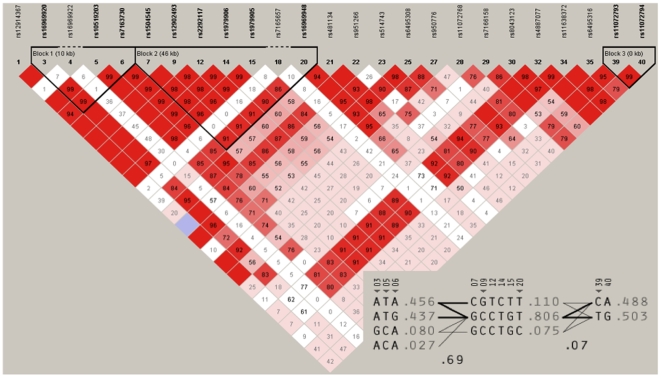
Haploview-generated LD patterns for 32 SNPs within the *CHRNA5/A3/B4* cluster in the Korean total sample. Pair-wise LD between all SNPs was evaluated using the Haploview program [30] with the option of determining haplotype blocks according to the criteria defined by Gabriel *et al*. [31]. The number in each box represents the *D'* value for each SNP pair.

The pair-wise *D'* values of 32 SNPs within *CHRNA5/A3/B4* were determined using the Haploview program [30]. On the basis of the block definition proposed by Gabriel *et al.* [31], we found three discernible haplotype blocks within the cluster in the total sample (Figure 1). The first block, with a size of about 10 kb, contains four SNPs between rs169669920 and rs7163730; the second block, with a size of 46 kb, contains 12 SNPs; the third block, with a size of 23 kb, contains only two SNPs — rs11072793 and rs11072794. Considering that (1) no haplotype block was detected within a genomic region of about 108 kb between rs481134 and rs6495316, and (2) the three discernible haplotype blocks did not correspond to the defined genes within this region, we decided to perform haplotype-based association analysis using a sliding window approach with a window size of three consecutive SNPs [32], as described in the following section.

### Haplotype-based association analysis

Using the Haplo.stats R statistics package [33], we performed haplotype-based association analysis for the three smoking-related phenotypes in both the total and male samples (Tables 3 and 4). In the total sample, we found five major haplotypes between SNPs rs16969948 in *CHRNA5* and rs6495316 in the intergenic region downstream from the 5′ end of *CHRNB4* that were significantly associated with SI under the additive or dominant model. Of these haplotypes, two were positively, and the remaining three were negatively, associated with SI, with P values ranging from 0.013 for the haplotype ACT, formed by rs950776-rs11072768-rs7166158, to 0.047, for the haplotype TCC, formed by rs16969948-rs481134-rs951266.

**Table 3:** Statistics estimates and P values for major haplotypes showing significant associations at the 0.05 level with at least one smoking-related phenotype under the additive and dominant models in the total sample.

SNP Number	SNP combinations	Haplo-type	Freq	SI			SQ			SC		
				Esti-mate	P	Global P value	Esti-mate	P	Global P	Esti-mate	P	Global P
18-19-20	rs16969948-rs481134-rs951266	TCC	0.76	−0.304^d^	**0.047^d^**	0.108^d^	−0.248^d^	0.087^d^	0.209^d^	−0.013^d^	0.928^d^	0.261^d^
19-20-21	rs481134-rs951266-rs514743	CCT	0.81	−0.326^d^	0.092^d^	0.233^d^	−0.147^a^−0.457^d^	**0.017^a^** **0.014^d^**	**0.006^a^** **0.050** ^d^	−0.105^d^	0.579^d^	0.299^d^
21-22-23	rs514743-rs6495308-rs950776	TGA	0.73	−0.081^a^	0.056^a^	0.263^a^	−0.111^a^	**0.038^a^**	0.134^a^	−0.072^a^	0.198^a^	0.324^a^
22-23-24	rs6495308-rs950776-rs11072768	GAA	0.70	−0.088^a^	**0.031^a^**	0.141^a^	−0.116^a^	**0.025^a^**	0.131^a^	−0.097^a^	0.073^a^	0.279^a^
23-24-25	rs950776-rs11072768-rs7166158	ACT	0.12	0.163^d^	**0.013^d^**	0.144^d^	0.176^d^	**0.033^d^**	0.200^d^	0.165^d^	0.06^d^	0.244^d^
24-25-26	rs11072768-rs7166158-rs8043123	CTC	0.15	0.118^a^	**0.034^a^**	0.103^a^	0.145^a^	**0.038^a^**	0.229^a^	0.136^a^	0.068^a^	**0.014^a^**
26-27-29	rs8043123-rs4887077-rs11638372	TGG	0.45	−0.051^d^	0.375^d^	0.327^d^	−0.172^d^	**0.020^d^**	**0.009^d^**	−0.038^d^	0.626^d^	0.344^d^
27-29-30	rs4887077-rs11638372-rs6495316	GGA	0.88	−0.634^d^	**0.028^d^**	0.073^d^	−0.531^d^	**0.044^d^**	0.125^d^	0.023^d^	0.931^d^	0.869^d^

(1) SI  =  smoking initiation; SQ  =  smoking quantity; SC  =  smoking cessation; (2) Significant associations at the 0.05 level before Bonferroni correction for multiple testing are given in bold; (3) Superscripts indicate the genetic model used: a  =  additive; d  =  dominant; (4) For each sample, age, sex, and geographic area were used as covariates.

**Table 4:** Statistics estimates and P values for major haplotypes showing significant associations at the 0.05 level with at least one smoking-related phenotype under the additive and dominant models in the male sample.

SNP Number	SNP combinations	Haplo-type	Freq	SI			SQ			SC		
				Esti-mate	P	Global P	Esti-mate	P	Global P	Esti-mate	P	Global P
18-19-20	rs16969948-rs481134-rs951266	TCC	0.77	−0.102^a^	**0.034^a^**	**0.023^a^**	−0.108^a^	0.069^a^	**0.003^a^**	−0.050^a^	0.401^a^	0.276^a^
19-20-21	rs481134-rs951266-rs514743	CCT	0.82	−0.173^a^	**0.020^a^**	**0.037^a^**	−0.186^a^	**0.005^a^**	**0.001^a^**	−0.011^a^	0.869^a^	0.529^a^
20-21-22	rs951266-rs514743-rs6495308	CTG	0.74	−0.114^a^−0.265^d^	**0.015^a^** **0.025^d^**	**0.008^a^** 0.137^d^	−0.124^a^−0.307^d^	**0.032^a^** **0.031^d^**	**0.003^a^** 0.158^d^	−0.077^a^	0.186^a^	0.303^a^
21-22-23	rs514743-rs6495308-rs950776	TGA	0.74	−0.118^a^	**0.011^a^**	0.071^a^	−0.134^a^	**0.019^a^**	0.060^a^	−0.082^a^	0.155^a^	0.411^a^
22-23-24	rs6495308-rs950776-rs11072768	GAA	0.70	−0.132^a^	**0.003^a^**	**0.038^a^**	−0.140^a^	**0.011^a^**	0.103^a^	−0.107^a^	0.054^a^	0.288^a^
23-24-25	rs950776-rs11072768-rs7166158	AAT	0.57	−0.101^a^	**0.018^a^**	**0.024^a^**	−0.116^a^	**0.029^a^**	0.112^a^	−0.072^a^	0.180^a^	0.161^a^
		ACT	0.12	0.168^a^0.238^d^	**0.009^a^** **0.001^d^**	**0.024^a^ 0.013^d^**	0.173^a^0.214^d^	**0.026^a^** **0.014^d^**	0.112^a^ 0.117^d^	0.162^a^0.210^d^	**0.043^a^** **0.019^d^**	0.161^a^ 0.154^d^
24-25-26	rs11072768-rs7166158-rs8043123	ATC	0.38	−0.094^a^−0.141^d^	**0.032^a^** **0.029^d^**	**0.010^a^** 0.073^d^	−0.047^a^	0.393^a^	0.208^a^	−0.050^a^	0.359^a^	**0.009^a^**
		CTC	0.15	0.157^a^0.149^d^	**0.010^a^** **0.035^d^**	**0.010^a^** 0.073^d^	0.159^a^	**0.031^a^**	0.208^a^	0.159^a^	**0.036^a^**	
26-27-29	rs8043123-rs4887077-rs11638372	CGG	0.53	−0.229^d^	**0.022^d^**	**0.035^d^**	−0.134^d^	0.128^d^	**0.021^d^**	0.122^d^	0.158^d^	0.369^d^

(1) SI  =  smoking initiation; SQ  =  smoking quantity; SC  =  smoking cessation; (2) Significant associations at the 0.05 level before Bonferroni correction for multiple testing are given in bold and those after Bonferroni correction for multiple testing are given in bold and underlined (corrected P value at a 0.05 significance level is 0.0125 under the assumption of a maximum of four major haplotypes for each SNP combination); (3) Superscripts indicate the genetic model used: a =  additive; d =  dominant; and (4) For each sample, age, and area were used as covariates.

For SQ, we found seven major haplotypes between SNPs rs16969948 in *CHRNA5* and rs6495316 in the intergenic region downstream from the 5′ end of *CHRNB4* that showed significant associations. Of these haplotypes, two were positively, and the remaining five were negatively, associated with SI, with a P value ranging from 0.014 for the haplotype CCT, formed by rs481134-rs951266-rs514743, to 0.044 for the haplotype GGA, formed by rs4887077-rs11638372-rs6495316. Although we performed identical association analyses for SC, we found no significant haplotypes in the total sample. By comparing significant haplotypes with SI and SQ, we found four haplotypes, GAA (rs6495308-rs950776-rs11072768), ACT (rs950776-rs11072768-rs7166158), CTC (rs11072768-rs7166158-rs8043123), and GGA (rs4887077-rs11638372-rs6495316), to be significantly associated with both phenotypes under identical genetic models, indicating that they contribute to the etiologies of these two behaviors, likely through a common uncharacterized mechanism.

Similarly, we performed haplotype-based association analysis for the male sample (Table 4). Altogether, we found that ten haplotypes between SNPs rs16969948 in *CHRNA5* and rs11638372 in the intergenic region downstream from the 5′ end of *CHRNB4* showed significant associations with at least one smoking-related phenotype. By comparing the haplotype-based association results of the total and male samples, we found six that were comparable in the two samples, except that the association signals appear to be much stronger in the male sample. For example, the P values of associations with SI, SQ, and SC for the haplotype ACT, formed by rs950776-rs11072768-rs7166158 under the dominant model, are, respectively, 0.001, 0.014, and 0.019 in the male sample and 0.013, 0.033, and 0.06 in the total sample. Very interestingly, we found that haplotypes ACT, formed by rs950776-rs11072768-rs7166158, and CTC, formed by rs11072768-rs7166158-rs8043123, were nominally significantly associated with all three smoking-related phenotypes in the male sample, suggesting that this region is a good target for further sequencing analysis with the hope of identifying variants that contribute to our observed association of the cluster with these smoking-related phenotypes.

### Interaction analysis of *CHRNA5/A3/B4* with SI, SQ, and SC

To further determine a genetic contribution of the *CHRNA5/A3/B4* cluster to the three smoking-related phenotypes, we performed a gene-gene interaction analysis for the total and male samples as we did previously for nAChR genes alpha 4 (*CHRNA4*) and beta 2 (*CHRNB2*) [34] and GABA-B receptor subunits 1 (*GABAB1*) and 2 (*GABAB2*) [35]. By using the generalized multifactor dimensionality reduction (GMDR) approach [36], we performed the interaction analysis for all possible combinations of two to six SNPs within *CHRNA5/A3/B4* for SI and SC in both the total and the male sample. Among these gene-gene interaction analyses, a significant result emerged only in the male sample for SI, with the best interaction model consisting of SNPs rs7163730 in *LOC123688*, rs6495308 in *CHRNA3* and rs7166158, rs8043123, and rs11072793 from the downstream region of *CHRNB4*; the corresponding P value was 0.011 (Table 5). Because of the limitation of the current GMDR version, which cannot handle ordinal phenotypes such as SQ, we could not carry out gene-gene interaction analysis on this particular phenotype in our samples. However, we did perform interaction analysis on SQ by employing a cumulative *logit* model, which revealed no significant interactions of SNPs located in this genomic region in affecting SQ.

**Table 5:** A significant interactive model for *CHRNA5/A3/B4* cluster with SI in KARE male sample on the basis of the prediction accuracy and the sign test P value.

No. ofLoci	Best Model	Prediction Accuracy	Sign Test (P)	CVC
5	*LOC123688*: rs7163730*CHRNA3*: rs6495308*Inter-genic region*: rs7166158, rs8043123, rs11072793	0.517	9 (0.011)	10/10

(1) SI  =  smoking initiation; (2) CVC  =  cross-validation consistency; and (3) In GMDR analysis, age and area were used as covariates.

## Discussion

In the current study, we examined genetic associations and epistatic variants in *CHRNA5/A3/B4* with SI, SQ, and SC in the total and male samples. Individual SNP-based association analyses revealed that seven SNPs within this region showed significant associations with at least one smoking behavior in either or both samples. Of these polymorphisms, rs951266 in *CHRNA5* showed the strongest association with SI (P = 0.006) and SQ (P = 0.001) and rs11072768 in *CHRNB4* with all the three smoking-related phenotypes in the total (SI: P = 0.015; SQ: P = 0.028; SC: P = 0.018) and male (SI: P = 0.001; SQ: P = 0.016; SC: P = 0.010) samples. Furthermore, we found multiple haplotypes between rs16969948 in *CHRNA5* and rs6495316 or rs11638372 in the intergenic region downstream from the 5′ end of *CHRNB4* that were associated significantly with the three smoking-related phenotypes in both the total and the male sample. Finally, considering the fact that the protein products of these nAChR subunit genes must assemble in order to form functional nAChRs, we also performed gene-gene interaction analysis on all SNPs in this genomic region and found the best interactive model involved five SNPs located in *LOC123688*, *CHRNA3*, and the intergenic region at the 5′ end of *CHRNB4* that influences SI in the male sample.

**Figure 2:**
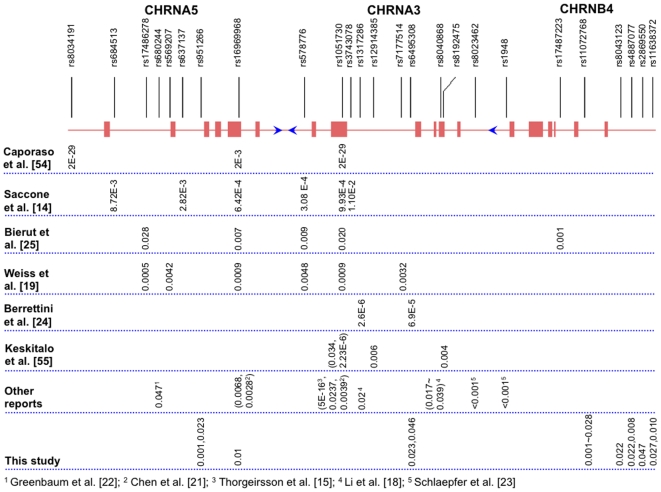
A summary of all reported SNPs that have been significantly associated with different smoking behaviors. Of these reported studies mentioned in the figure, most were investigated on nicotine dependence except that the study authored by Keskitalo et al. [55] was on serum cotinine level and that by Schlaepfer et al. [23] was on smoking initiation.

To assess ND, various scales have been developed, which include the Diagnostic and Statistical Manual of Mental Disorders (DSM)-IV criteria [4], Fagerström Test for ND (FTND) [37], HSI [38], the Nicotine Dependence Syndrome Scale (NDSS) [39], and the Wisconsin Inventory of Smoking Dependence Motives (WISDM-68) [40], to name a few. Among them, FTND is the most widely used instrument, primarily because of its succinctness and ease of administration [41]. The FTND score is commonly treated as a continuous variable, and an FTND ≥4 is typically defined as highly dependent [28], [42]. CPD, the most commonly used consumption measure [38], is both highly heritable [7] and predictive of ND [24], [38]. SQ is an ordinal variable based on CPD, which provides a simple, quantified index of the amount of cigarette consumption. It should be noted that SQ and FTND are correlated but different psychometric measures of smoking behavior. To date, associations of variants in the *CHRNA5/A3/B4* cluster with smoking have been detected for both SQ and FTND phenotypes, but signals appear to be stronger for SQ than for FTND (Figure 2), which is also true for linkage study on ND based on a review of more than 20 independent studies [43].

There are several strengths of this study. First, although significant associations of variants within the *CHRNA5/A3/B4* cluster with smoking have been replicated in multiple independent samples, almost all these studies were concentrated on smoking dependence in samples of European origin [14], [18], [19], [20], [21], [22], [23], [24], [25]. Very recently, relatively weak associations of some variants of this gene cluster with ND were reported in two African American samples [18], [28]. Among the 76 SNPs investigated by Saccone et al. [28], eight showed nominally significant association with ND (P value  = 0.0147∼0.0443), with rs16969968 in *CHRNA5* being the strongest, at a P value of 0.0147. Similarly, in a prior study [18], we analyzed 22 SNPs within the gene cluster and found that only rs8040868 in *CHRNA3* showed a nominally significant association with ND (P = 0.017∼0.039). However, none of these associations survived correction for multiple testing in the two African American samples [18], [28]. Although there were two studies investigating potential associations of variants in the gene cluster with LC and smoking in Chinese [44] and Japanese [45] subjects, no previous reports have examined the involvement of variants of this cluster with smoking behavior in a strictly Asian sample. Therefore, this study represents the first report on a potential association of the *CHRNA5/A3/B4* cluster with smoking behaviors in an Asian population. By identifying risk alleles for smoking and LC and comparing their frequencies in different ethnic populations, it is easy to see that they are quite different, which further underscores that different variants in this genomic region contribute importantly to variations in disease risks among different ethnic populations [12].

Second, most previous association studies of the *CHRNA5/A3/B4* cluster were focusing on ND or related measures [14], [15], [19], [24], [25], although a few studies were focused on SI [23], [27] or SC [26], [46], [47]. Given the significant overlap of genetic underpinnings among these three smoking-related phenotypes [48], [49], it is of great interest to investigate these phenotypes systemically in one sample. This is especially true for the *CHRNA5/A3/B4* cluster, as it has been associated with ND in several reports, especially in samples of European origin [14], [18], [19], [20], [21], [22], [23], [24], [25]. However, association of the variants of this cluster with SI and SC has not been well established. By analyzing the three major smoking-related phenotypes together in the Korean sample, we found significant associations of some variants in this cluster with the three smoking-related phenotypes at both the individual SNP and the haplotype levels.

Third, in this study, we not only performed association analyses of this region with the three major smoking-related phenotypes at the SNP and haplotype levels, as in most reported association studies of the region with smoking, but also performed extensive gene-gene interaction analysis exclusively in this region. This is important in that: (1) all nAChR subunits except alpha 7 must assemble under appropriate compositions in order to form functional receptors; and (2) it is still undetermined whether other uncharacterized genes of this region contribute to smoking-related phenotypes [12]. Our gene-gene interaction results in the male sample indicate clearly that some variants exist within the region between rs7163730 in *LOC123688* and rs11072793 in the intergenic region downstream from the 5′ end of *CHRNB4* that are contributing to SI through gene-gene interactions. This appears consistent with findings from our recent study showing an association of the region with ND in European American and African American samples [18]. This has been the case for several other genes also. For example, we recently detected a significant interaction between variants of *CHRNA4* and *CHRNB2* in affecting ND, such that *CHRNB2* contributes significantly to the etiology of ND together with *CHRNA4* through gene-gene interaction [34]. Similarly, in another study, we found that *GABAB1* contributes to ND through its interaction with *GABAB2*, although no significant association was observed for *GABAB1* with ND directly [35].

There exist several limitations to this study. First, we did not consider Bonferroni correction for all possible multiple testing, primarily because we consider this study a replication of a positive association of this cluster with ND in an independent sample. A similar approach has been adopted by other researchers [25]. If we include correction for multiple testing, most detected associations of variants within this cluster with SI, SQ and SC at both the individual SNP and haplotype levels become non-significant, with the exceptions that rs951266 in *CHRNA5* remained significant for SQ and rs11072768 in *CHRNB4* for SI in the male sample (see Table 3). Similarly, we found that associations of four major haplotypes with SI and two major ones with SQ remained significant after Bonferroni correction for multiple testing of major haplotypes (Table 4). However, no significant associations of any SNPs or haplotypes with SC remained after Bonferroni correction for multiple testing. Also, we did not correct for our testing of two genetic models and three phenotypes, as they are highly related, which violates the assumption of independence for Bonferroni correction. Because of the aforementioned concerns, to some extent, we consider this study explorative, and more replication in Korean or other Asian samples is greatly needed. Second, although we defined the three smoking-related phenotypes on the basis of all the related information collected from each smoker, we are not fully convinced this is the best sample for investigation of this gene cluster and smoking behaviors, as this work was not designed originally as a genetic study on smoking, and the information collected from each smoker is limited. For example, SQ was assessed by CPD instead of ND determined by the FTND or DSM-IV criteria commonly used in other studies. Also, there was no SQ information (i.e., a 5-category ordinal trait based on CPD) for light or occasional smokers, although we do know they smoked only when they were in specific social circumstances such as during a party or gathering with their friends. Further, the SC status was based on individual self-reports, which were not biochemically verified, and this might lead to potential bias in phenotyping. Nevertheless, we do not feel this bias would greatly affect our results, as the definitions used in the current study have been used in other studies of this type as well [27], [43]. This is especially true for SQ, as it produces significantly more positive findings than any other smoking measures for ND in both linkage and association analyses [43]. Third, we investigated the association of this cluster with three smoking-related phenotypes only in the Korean total and male samples. We did not perform a similar analysis of the female sample because of the small sample size attributable to the small percentage of female smokers (3%), which is the case for many other Asian countries such as China (∼4% of Chinese women aged 15 years or older are smokers [50]). Thus, we could not determine whether this gene cluster has any significant impact on smoking behaviors in Korean women. Finally, the number of informative SNPs genotyped within this region was limited. To have a better coverage for these samples, more SNPs with a high density are greatly needed for further analysis of this cluster in relation to smoking-related phenotypes.

As shown in Table S1, the allele frequencies of an array of genetic variants (e.g., rs7168796, rs16969922, and rs1979906, to name a few) located in *CHRNA5/A3/B4* differ dramatically across ethnic and geographical populations (i.e., the Caucasian [CEU], African [YRI], and Asian [CHB, JPT, and Korean] samples). Therefore, this study provides an essential basis for genetic association analysis by comparing different frequencies of SNPs of this region between smoking and non-smoking populations to examine whether such associations are consistent in different ethnicities. Indeed, different genotype frequencies have been compared in smoking and non-smoking populations in different ethnic groups for several polymorphisms of CYP2A6 [51] as well as a 44-bp insertion/deletion polymorphism of the serotonin transporter (5-HTT) gene [52], and such studies may shed new light on what molecular variants have diverging effects, and what molecular variants have converging effects in populations of different ancestries. This approach is termed “cross-population contrast mapping” based on the hypothesis that important biological mechanisms underlying a disease of interest are shared by various human populations, although differences in allele frequencies at risk loci could result in different prevalences in different populations [53]. Because smoking is a significant risk factor for LC, and the association of *CHRNA5/A3/B4* with smoking or LC risk had been reported in many other populations, the present work should contribute to establishing that different polymorphic *CHRNA5/A3/B4* patterns associate with the physiology of smoking and LC in ethnically and geographically different populations.

**Figure 3:**
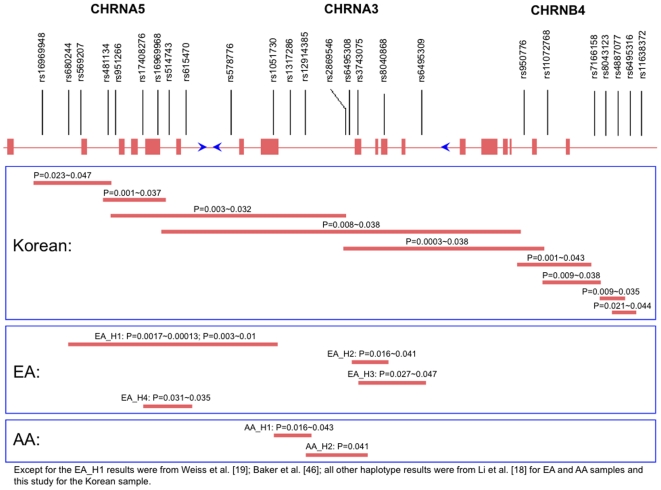
A summary of all reported haplotypes that have been significantly associated with different smoking behaviors in the AA, EA and Korean samples.

To compare the association signals detected by this study with those detected in other populations from previous studies, we have summarized both individual SNP and haplotype results in Figures 2 and 3, respectively. To our knowledge, approximately 25 SNPs in this region are reported to be associated with smoking-related and LC phenotypes. Among the 7 SNPs found to be nominally associated with SI, SQ, and SC, rs6495308 in *CHRNA3* was also found associated with CPD in two European American samples [24]. All the remaining 6 SNPs (1 in *CHRNA5*, 1 in *CHRNB4*, and 4 in the intergenic regions) were unique to this Korean sample. It would be of great interest to individually genotype additional SNPs in this region in the sample, especially rs1051730 and rs16969968, which showed unequivocally significant association signals with SQ or CPD in several previous studies [15], [54], [55]. Recently, Le Marchand et al. [56] used urinary biomarkers to test whether rs1051730 and rs16969968 are associated with a higher level of nicotine and exposure to tobacco-specific carcinogenic substances per cigarette dose in a Hawaii study of 583 men and women of European, Japanese, or Native Hawaiian ancestry who were long-term smokers of more than 10 cigarettes per day. Although the T of rs1051730 and A of rs16969968 alleles were less common in Japanese Americans (3%) and Native Hawaiians (∼19%) than in European Americans (34%), those investigators found that carriers of T of rs1051730 or A of rs16969968 extract a greater amount of nicotine (P = 0.004 and 0.003, respectively) and those A carriers of the rs16969968 had a higher internal dose of total 4-(methylnitrosamino)-1-(3-pyridyl)-1-butanol (P = 0.03) per cigarette than non-carriers. In another study with a sample size of ∼17,300 subjects from five LC studies and four upper aerodigestive tract cancer studies, Lips et al. [57] revealed no association between rs16966968 and SI or SC, age at SI, or age at SC. However, when cancer cases and controls were combined (after adjustment for case/control status), the adjusted mean difference between the two homozygote genotypes was 1.2 CPD (P<0.0001). Moreover, the rs16969968 genotype was associated with LC in both former (P<0.0001) and current (P<0.0001) smokers, and a marginally significant trend was observed in never smokers (P = 0.07).

At the haplotype level, in the three long-term EA smoker cohorts recruited in Utah, Wisconsin, and by the NHLBI Lung Health Study, Weiss et al. [19] found that a 5-SNP haplotype H_A_ (CCAGA) formed by rs680244-rs569207-rs16969968-rs578776-rs1051730 (denoted EA_H1), had a risk effect on high FTND score (>6) compared with low FTND score (<4) (OR = 1.50, 95% CI: 1.21, 1.86, P = 1.3×10^−4^), and the 5-SNP haplotype H_C_ (CTGAG) formed by the same SNPs as in H_A_ had a protective effect on high ND (OR = 0.66, 95% CI: 0.52, 0.85, P = 1.07×10^−3^). Such findings corroborated for ND assessed by Primary Dependence Motives (PDM) score [46]. Further, our previous study in the Mid-South Tobacco Family (MSTF) cohort revealed 3 additional haplotypes in EAs [18], formed by rs6495308-rs3743075-rs8040868 (EA_H2), rs3743075-rs8040868-rs6495309 (EA_H3), and rs17408276-rs16969968-rs615470 (EA_H4), that were associated with smoking-related phenotypes. In comparison with EA_H1, although EA_H4 was physically encompassed by EA_H1, and they share 1 SNP (i.e., rs16969968), EA_H2 and EA_H3, which have 2 overlapping SNPs (i.e., rs3743075 and rs8040868), were physically separate from EA_H1. Also in the MSTF cohort, we detected two AA haplotypes formed by rs1317286-rs12914385-rs2869546 (AA_H1) and rs2869546-rs6495308-rs3743075 (AA_H2), which share 1 SNP (i.e., rs2869546) (Figure 3). Comparing the detected haplotypes in AAs and EAs, AA_H1 does not physically overlap with any of the 4 EA haplotypes, and AA_H2 shares 2 SNPs (i.e., rs6495308 and rs3743075) with EA_H2, as well as 1 SNP (i.e., rs3743075) with EA_H3. Although the overall physical region spanned by the 9 overlapping Korean haplotypes identified in this study from rs16969948 to rs6495316 does encompass all 4 EA haplotypes (i.e., EA_H1, EA_H2, EA_H3, and EA_H4) as well as both AA haplotypes (i.e., AA_H1 and AA_H2), these 9 Korean haplotypes do not share any SNPs with EA_H1 EA_H3, EA_H4 or AA_H1. Nevertheless, 3 Korean haplotypes (Korean_H3, Korean_H4, and Korean_H5) do share an SNP (i.e., rs6495308) with EA_H2 and AA_H2. Overall, the Korean haplotypes provide some support for the importance of the EA and AA haplotypes previously associated with smoking-related phenotypes However, because the SNP compositions differ across EA, AA and Korean haplotypes, the haplotype association signals are not directly comparable. To get a clearer picture of cross-population haplotypic effects, it is essential to examine the effects of haplotypes with the same SNP combinations on smoking behavior across different ethnic populations given that haplotype frequencies differ so dramatically in populations of different ancestries.

The problem of multiple hypothesis testing in biomedical studies is an important yet complex issue that needs to be considered carefully [58]. For complex traits such as smoking behavior, variants in genes of multiple biological pathways are likely to be involved in producing the phenotype through their mutual interactions and interactions with environmental factors. Therefore, genetic effects of common variants for smoking behavior are often weak secondary to genetic heterogeneity, variable expressivity, and low penetrance; and exactly how to correct for multiple testing remains a debatable and profound topic. For example, for gene-based studies such as this study focusing on the *CHRNA5/A3/B4* cluster, Neale and Sham [59] have emphasized the importance of replication rather than the sole interest of detecting association signals with very low P values, and the authors have challenged the need for correcting for multiple testing in this specific scenario. Conversely, from a statistical viewpoint, when a data set is tested in multiple angles, the threshold for statistical significance should be adjusted to reduce the inflated type I error. To correct for multiple testing, as suggested by Feise [60], a prudently chosen balance needs to be reached for a study's statistical significance in consideration of multiple factors such as the magnitude of the genetic effect, biological function(s) of the marker(s) of interest, the study's quality, as well as the collective supportive evidence of the genetic locus from other independent studies [60]. Specifically, in this study, we applied the Bonferroni correction procedure for the number of SNPs analyzed within each sample for a given phenotype, but we did not correct for the number of smoking-related phenotypes (i.e., SI, SQ, and SC), the number of genetic models employed (i.e., recessive, additive, and dominant), or the number of study samples (i.e., total and male sample). The rationales underlying our decisions are that the smoking-related phenotypes are highly inter-related, the male smokers constitute the predominant fraction of all smokers in the total sample, and the three genetic models cannot be treated as totally independent. Therefore, given that the variants in *CHRNA5/A3/B4* have been associated with smoking behavior in various independent samples of European origin, the correction employed was a balanced decision between the application of a relatively stringent Bonferroni correction and a precaution in guarding against an over-correction that could result in unintended losses of power and valuable information [61], [62].

In sum, this is the first genetic study aimed at investigating any association of the variants of the *CHRNA5/A3/B4* cluster with SI, SQ, and SC in Korean smokers. Despite the caveats regarding correction for multiple testing, our replication of prior reported significant associations of *CHRNA5/A3/B4*, combined with the convergent biological data implicating the functional roles of nAChR subunit genes residing in this cluster in addiction, strengthens the notion that the multiple nominally significant association signals we detected at both the SNP and haplotype levels in the Korean sample are true positives. Moreover, our study is the first to show that the nominally significant association signals have extended beyond the 5′ end of *CHRNB4* to the flanking intergenic region. Further, we found that the associations of this cluster region with SI and SQ phenotypes appeared to be stronger than those with the SC phenotype in Koreans smokers. Finally, we provided evidence for nominally significant interactions among variants studied in affecting SI in the male sample. Although these findings are novel and encouraging, they do need to be confirmed in larger, independent studies of subjects with Asian ancestry.

## Materials and Methods

### Ethics statement

Informed written consent was obtained in advance from all participants using a form approved by all participating Institutional Review Boards. The study likewise was approved by the Institutional Review Boards of the National Institute of Health of Korea, Seoul National University, and the University of Virginia and was in accordance with the principles of the Helsinki Declaration II.

### Subjects and genotyping

The relevant information for the subjects in the current study has been reported [63]. Briefly, samples from the 10,038 participants in the Korea Association Resource (KARE) Project were obtained in two recruiting areas, Ansung and Ansan, in South Korea. Participant ages ranged from 40 to 69 years. Genomic DNA was available for 10,004 participants and was genotyped with the Affymetrix Genome-Wide Human SNP Array 5.0. Genotypes were called with Bayesian Robust Linear Modeling using the Mahalanobis Distance (BRLMM) algorithm. Those samples with low call rates (N = 401), contamination (N = 11), sex inconsistencies (N = 41), cryptic relatedness (N = 608), or serious concomitant illness (N = 101) were removed. After all the samples had been filtered with this standard quality control procedure [63], 8,842 individual samples remained for the current study (4,183 males and 4,659 females).

Although we had genotyping data available for other SNPs in these samples, in this study, we focused only on the *CHRNA5/A3/B4* region of chromosome 15, as this region has received much attention for its association with smoking and LC [14], [15], [16], [17], [24]. According to the genomic locations, 36 SNPs were selected. Of them, 32 met our inclusion criteria for this study with a satisfying Hardy-Weinberg equilibrium P value >10^−6^, MAF >0.01, and genotype call rates >95%. The 36 SNPs are described in Table 1.

### Definition of smoking-related phenotypes

On the basis of the survey questionnaire, from which information on smoking status (i.e., never smoker, former smoker, occasional or light smoker, and habitual smoker) and CPD for the habitual smokers were drawn, three smoking-related phenotypes were defined: SI, SQ, and SC. Regarding SI, the first measure (called SI-1) was defined as a binary trait comparing “never smoked” and “having regular smoking experiences,” and the second measure (called SI-2) was defined as an ordinal trait with four categories: never, former, light, and habitual. Because the association results for these two SI measures appear to be similar, in this communication, we show them together under the SI phenotype. The SQ phenotype was defined as an ordinal trait with five categories (1–5) according to CPD: non-smoking, <10 CPD, 11 to 20 CPD, 21 to 30 CPD, and >31 CPD. Such assessment of SQ not only has been commonly used in the literature but also is the most productive in terms of positive findings [43]. Finally, the SC phenotype was defined as a binary trait comparing “former smoking” and “current smoking.”

### Association analysis

Statistical analysis was performed using PLINK [29] and R software. For the binary phenotypes, SI-1 and SC, the association tests were performed using logistic regression analysis, with age, sex, and geographic area of recruitment as covariates under the additive and dominant models. The cumulative *logit* model was fit to the ordinal phenotypes SI-2 and SQ [64]. LD analysis was performed using Haploview software [30] with the option of determining haplotype blocks according to the definitions proposed by Gabriel *et al*. [31]. Haplotypes were reconstructed using SNPs within the genomic region containing the *CHRNA5/A3/B4* cluster with sliding window sizes of 3 to 5. Haplotype-based association analysis was carried out with the haplo.stats R statistics package [33] under additive and dominant models, adjusting for age, sex, and geographic area. As we consider this study a replication of reported significant association of variants of this gene cluster with smoking behavior in an independent sample [14], [15], [16], [17], [24], we did not correct for multiple comparisons for the two genetic models and three smoking-related phenotypes, primarily because they are related, when we interpreted our findings following the concept and approach used by other researchers [25]. However, we do recognize a potential limitation of this approach, which is addressed in the Discussion section herein.

### Interaction analysis of variants in the *CHRNA5/A3/B4* cluster

For the interaction analysis of variants in the *CHRNA5/A3/B4* region, both logistic regression and generalized multifactor dimensionality reduction (GMDR) analyses [36] were performed for binary traits (SI-1 and SC), whereas the cumulative *logit* model was applied to the ordinal phenotypes (SI-2 and SQ). The logistic regression model and cumulative *logit* model included the main effects of SNPs, interaction effects among the SNPs, as well as adjusting covariates. The log-likelihood ratio test was performed for testing the joint significance of SNP main and interaction effects. The GMDR analysis was carried out only for the binary traits, as GMDR analysis is not applicable to ordinal traits. Age, sex, and recruiting area were considered as adjusting covariates for all interaction analyses. The best SNP combination for all two- to six-locus models was selected via the prediction rate.

## Supporting Information

Table S1 Positions, nucleotide variations, and allele frequencies for SNPs on chromosome 15.
(0.09 MB DOC)
