# Supplementary material for: Associations of Variants in CHRNA5/A3/B4 Gene Cluster with Smoking Behaviors in a Korean Population
Source: PLoS One. 2010 Aug 16;5(8):e12183. doi: 10.1371/journal.pone.0012183 (PMC2922326; doi:10.1371/journal.pone.0012183)
Supplement: Table S1 — Positions, nucleotide variations, and allele frequencies for SNPs on chromosome 15. (0.09 MB DOC) [file pone.0012183.s001.doc]

**Table S1:** Positions, nucleotide variations, and allele frequencies for SNPs on chromosome 15

| Gene | Marker number | dbSNP ID | Chrom. Pos. | Major/Minor Alleles | SNP Location | MAF  (CEU)* | MAF  (YRI)* | MAF  (CHB)* | MAF  (JPT)* | MAF  (KARE Total) | MAF  (KARE Men) |
| --- | --- | --- | --- | --- | --- | --- | --- | --- | --- | --- | --- |
| *LOC123688* | 1 | rs12914367 | 76582087 | A/G | Upstream  (5000bp) | 0.000 | 0.325 | 0.011 | 0.078 | 0.01 | 0.01 |
| *LOC123688* | 2 | rs7168796 | 76587549 | T/C | Intron | 0.000 | 0.200 | 0.000 | 0.000 | 0.00 | 0.00 |
| *LOC123688* | 3 | rs16969920 | 76590778 | A/G | Intron | 0.000 | 0.093 | 0.045 | 0.056 | 0.08 | 0.08 |
| *LOC123688* | 4 | rs16969922 | 76592844 | G/C | Intron | 0.000 | 0.220 | 0.000 | 0.000 | 0.01 | 0.01 |
| *LOC123688* | 5 | rs10519203 | 76601101 | A/G | Intron | 0.433 | 0.300 | 0.091 | 0.067 | 0.11 | 0.11 |
| *LOC123688* | 6 | rs7163730 | 76601736 | A/G | Intron | 0.208 | 0.183 | 0.456 | 0.489 | 0.44 | 0.44 |
| *LOC123688* | 7 | rs1504545 | 76605526 | G/C | Intron | 0.358 | 0.100 | 0.178 | 0.078 | 0.11 | 0.12 |
| *LOC123688* | 8 | rs952215 | 76606208 | T/C | Intron | 0.358 | 0.100 | 0.178 | 0.080 | 0.10 | 0.10 |
| *LOC123688* | 9 | rs12902493 | 76606330 | C/G | Intron | 0.358 | 0.100 | 0.178 | 0.078 | 0.11 | 0.12 |
| *PSMA4* | 10 | rs3813571 | 76619847 | G/T | 5′-UTR | 0.358 | 0.108 | 0.186 | 0.078 | 0.14 | 0.14 |
| *PSMA4* | 11 | rs2292117 | 76621744 | G/A | Intron | 0.358 | 0.167 | 0.178 | 0.078 | 0.11 | 0.12 |
| *PSMA4* | 12 | rs4887063 | 76626770 | T/C | Intron | 0.364 | 0.178 | 0.178 | 0.089 | 0.10 | 0.10 |
| *Intergenic region* | 13 | rs1979906 | 76629344 | T/C |  | 0.373 | 0.175 | 0.178 | 0.078 | 0.10 | 0.11 |
| *Intergenic region* | 14 | rs1979905 | 76629429 | C/A |  | 0.367 | 0.175 | 0.178 | 0.078 | 0.12 | 0.12 |
| *Intergenic region* | 15 | rs1504547 | 76630371 | C/T |  | 0.000 | 0.075 | 0.000 | 0.000 | 0.00 | 0.00 |
| *CHRNA5* | 16 | rs11636732 | 76641978 | T/G | Upstream  (5000bp) | 0.018 | 0.008 | 0.000 | 0.011 | 0.01 | 0.01 |
| *CHRNA5* | 17 | rs7165657 | 76648406 | C/G | Intron | 0.000 | 0.192 | 0.000 | 0.000 | 0.00 | 0.00 |
| *CHRNA5* | 18 | rs16969948 | 76651841 | A/G | Intron | 0.000 | 0.200 | 0.044 | 0.033 | 0.08 | 0.08 |
| *CHRNA5* | 19 | rs481134 | 76664618 | G/A | Intron | 0.364 | 0.225 | 0.144 | 0.111 | 0.14 | 0.14 |
| *CHRNA5* | 20 | rs951266 | 76665596 | G/A | Intron | 0.417 | 0.117 | 0.033 | 0.011 | 0.02 | 0.02 |
| *CHRNA5* | 21 | rs514743 | 76671282 | A/T | Intron | 0.325 | 0.200 | 0.167 | 0.159 | 0.16 | 0.15 |
| *CHRNA3* | 22 | rs6495308 | 76694711 | T/C | Intron | 0.212 | 0.342 | 0.273 (T) | 0.222 (T) | 0.26 (T) | 0.25 (T) |
| *CHRNB4* | 23 | rs950776 | 76713073 | T/C | Intron | 0.300 | 0.058 | 0.156 | 0.136 | 0.13 | 0.13 |
| *CHRNB4* | 24 | rs11072768 | 76716533 | G/T | Intron | 0.183 | 0.325(G) | 0.211(G) | 0.178(G) | 0.20(G) | 0.20(G) |
| *Intergenic region* | 25 | rs7166158 | 76735808 | A/T |  | 0.100 | 0.149 | 0.227 | 0.186 | 0.20 | 0.20 |
| *Intergenic region* | 26 | rs8043123 | 76760448 | C/T |  | 0.233 | 0.125 | 0.489 | 0.389 | 0.45 | 0.45 |
| *Intergenic region* | 27 | rs4887077 | 76765419 | C/T |  | 0.442 | 0.017 | 0.033 | 0.011 | 0.02 | 0.02 |
| *Intergenic region* | 28 | rs2869550 | 76768056 | T/C |  | 0.000 | 0.000 | 0.400 | 0.400 | 0.47 | 0.48 |
| *Intergenic region* | 29 | rs11638372 | 76770614 | C/T |  | 0.442 | 0.017 | 0.033 | 0.011 | 0.02 | 0.02 |
| *Intergenic region* | 30 | rs6495316 | 76773156 | A/T |  | 0.017 | 0.342 (A) | 0.044 | 0.144 | 0.10 | 0.10 |
| *Intergenic region* | 31 | rs8041651 | 76773813 | T/G |  | 0.000 | 0.017 | 0.302 | 0.330 | 0.16 | 0.17 |
| *Intergenic region* | 32 | rs11633170 | 76791697 | T/C |  | 0.183 | 0.200 (T) | 0.321 | 0.488 (T) | 0.34 | 0.35 |
| *Intergenic region* | 33 | rs11072793 | 76793497 | A/G |  | 0.242 | 0.200 (A) | 0.467 | 0.411 (A) | 0.50 | 0.50 |
| *Intergenic region* | 34 | rs11072794 | 76793637 | C/T |  | 0.242 | 0.150 (C) | 0.456 | 0.443 (C) | 0.49 | 0.49 |
| *Intergenic region* | 35 | rs2869560 | 76810875 | G/C |  | 0.000 | 0.155 | 0.000 | 0.000 | 0.00 | 0.00 |
| *Intergenic region* | 36 | rs2219939 | 76816778 | A/G |  | 0.246 | 0.076 (A) | 0.122 (A) | 0.091 (A) | 0.13 (A) | 0.12 (A) |

*Based on the allele frequency from the NCBI database. CEU = CEPH (Utah residents with ancestry from northern and western Europe); YRI = Yoruba in Ibadan, Nigeria; CHB = Chinese Han in Beijing, China; and JPT = Japanese in Tokyo, Japan.
